# Anesthesia effects of different doses of fospropofol disodium for painless colonoscopy diagnosis and treatment

**DOI:** 10.3389/fmed.2025.1562592

**Published:** 2025-03-14

**Authors:** Fangli Yue, Xinyuan Shi, Ruoyi Ji, Yaxin Wei, Hongyi Xiao, Huan Zhang, Min Fu, Fanceng Ji

**Affiliations:** ^1^Department of Anesthesiology, Weifang People’s Hospital, Weifang, China; ^2^School of Anaesthesiology, Shandong Second Medical University, Weifang, China; ^3^School of Psychology and Cognitive Sciences, Peking University, Beijing, China

**Keywords:** fospropofol disodium, alfentanil, colonoscopy, anesthetic effect, intravenous anesthesia

## Abstract

**Purpose:**

To explore the clinical anesthesia effect of different doses of fospropofol disodium for painless colonoscopy.

**Patients and methods:**

A total of 69 patients undergoing colonoscopy under general intravenous anesthesia were included in this study. They were divided into three groups: fospropofol disodium 10 mg/kg group (P1 group, *n* = 23), fospropofol disodium 12.5 mg/kg group (P2 group, *n* = 23), and fospropofol disodium 15 mg/kg group (P3 group, *n* = 23). All patients were first injected with 5 μg/kg of alfentanil intravenously at the time of anesthesia induction, 1 min later, group P1, group P2 and group P3 were given 10 mg/kg, 12.5 mg/kg and 15 mg/kg of fospropofol disodium, respectively. The success rate of the first sedation, the time of sedation start, the time of awakening, hemodynamic changes and adverse reactions were recorded.

**Results:**

The success rate of first sedation in the P2 and P3 groups was significantly higher than that in the P1 group (*p* < 0.05). The onset time of sedation was significantly shorter in the P2 and P3 groups than in the P1 group (*p* < 0.05). The awakening time of the P2 group and the P3 group (9 min vs. 7 min) was significantly longer than that of the P1 group (5 min) (*p* < 0.05). The incidence of hypotension in the P3 group was significantly higher than that in the P1 and P2 groups (*p* < 0.05). At T2, the MAP of the P3 group decreased significantly compared with the P1 and P2 groups (*p* < 0.05). There were no significant differences in adverse reactions such as injection pain, abnormal sensation/itching between the three groups (*p* > 0.05).

**Conclusion:**

In painless colonoscopy, fospropofol disodium 12.5 mg/kg combined with alfentanil 5 μg/kg has a high success rate of first-time sedation and low hemodynamic impact, which has some clinical advantages.

**Clinical trial registration:**

ChiCTR2400090788.

## 1 Introduction

With the promotion of comfortable diagnosis and treatment, the proportion of patients receiving anesthesia and sedation in colonoscopy is increasing (1). Colonoscopy diagnosis and treatment under anesthesia can improve the comfort of patients, reduce the occurrence of accidental injury during the examination, and improve the satisfaction of endoscopic operators (2). Fospropofol disodium is the first water-soluble prodrug of propofol in China. After being metabolized into the active substance propofol in the body, it exerts sedative or anesthetic effects on postsynaptic neurons by enhancing GABA receptor-mediated chloride influx and inhibiting NMDA receptor-mediated calcium influx (3–5). At present, the domestic research on fospropofol disodium mainly focuses on the induction and maintenance of general anesthesia. Domestic scholars recommend that the first loading dose of fospropofol disodium for tracheal intubation general anesthesia induction is 10 ~ 15 mg/kg (6). In foreign countries, fospropofol disodium is mainly used for monitored anesthesia care (MAC) in bronchoscopy and colonoscopy. The recommended effective dose is 6.5 mg/kg, and the maximum dose is 12.5 mg/kg. The maximum dose of 12.5 mg/kg will cause loss of consciousness in about 4 min (7). However, the appropriate dose of fospropofol disodium for deep sedation in painless colonoscopy has not been studied. This study intends to compare the clinical anesthetic effects of different doses of fospropofol disodium for painless colonoscopy diagnosis and treatment, and provide reference for clinical practice.

## 2 Materials and methods

This is a randomized controlled study to compare the clinical anesthetic effects of different doses of fospropofol disodium for painless colonoscopy. In this study, a total of 69 patients from Weifang People’s Hospital who were to undergo painless colonoscopy were selected as the research subjects. The study was approved by the Medical Ethics Committee of Weifang People’s Hospital [KYLL20240914-1] and the Chinese Clinical Trials Registry [ChiCTR2400090788], and informed consent was obtained from patients. This study was carried out in accordance with the principles of the Declaration of Helsinki. All patients obtained written informed consent preoperatively.

### 2.1 Patient inclusion criteria

Patients who were to undergo painless colonoscopy were selected, aged 18 ~ 65 years old, with a body mass index (BMI) of 18 ~ 30 kg/m^2^, ASAI~II and the examination time was expected to be 10 ~ 30 min. Exclusion criteria: patients with allergies to the use of drugs in the study, apnea-sleep syndrome, severe respiratory system, cardiovascular system diseases, neuromuscular system diseases, psychiatric diseases, as well as liver and kidney function, coagulation insufficiency.

### 2.2 Randomization and blinding

Patients were randomly divided into P1, P2 and P3 in a 1:1:1 ratio, and the randomization sequence was created by a person who was not involved in the trial implementation, and the randomization sequence was generated by a remote computer to ensure that the assignment was hidden. Participants and outcome assessors are blinded to group assignments, and anesthesia providers cannot be blinded due to significant differences in anesthetic techniques.

### 2.3 Methods of anesthesia

Before colonoscopy, patients should routinely prepare their bowels, fasting for at least 8 h and not being able to drink water for 4 h. After entering the room, the patient was in the left decubitus position, the peripheral veins of the upper limbs were opened, nasal catheter to snuff oxygen at 6 L/min, and connected to a monitor (Model: BeneVision N15, Shenzhen Mindray Biomedical Electronics Co., Ltd.), the basic vital signs such as NIBP, HR, SpO_2_, and RR were routinely monitored. Slowly intravenous injection of alfentanil 5 μg/kg within 30 s, 1 min later, group P1, group P2 and group P3 were given 10 mg/kg, 12.5 mg/kg and 15 mg/kg of fospropofol disodium, respectively. The patient’s sedation level was assessed using the Modified Observer’s Assessment of Alertness/Sedation (MOAA/S) scale. The endoscopic procedure was initiated once the patient’s eyelash reflex disappeared and the MOAA/S ≤ 1. If the MOAA/S score remained >1 5 mins after administering the study drug fospropofol disodium, it was considered a sedation failure. In such cases, propofol at a dose of 0.5 to 1 mg/kg was used as a rescue medication until the MOAA/S ≤ 1. During the examination, additional doses of fospropofol disodium at 2 mg/kg could be administered based on the patient’s sedation status (MOAA/S ≥ 2). If the patient is intravenously given 0.5 mg of atropine when HR is <50 times/min, ephedrine 6 mg intravenously when the mean arterial pressure (MAP) is <65 mmHg, and Jaw support is <90% when SpO_2_ is 90%, to improve airway obstruction or mask-assisted ventilation. The same attending physician in gastroenterology is responsible for endoscopy, and the same attending physician in anesthesiology is responsible for anesthesia.

### 2.4 Measurements

Primary outcome: the success rate of first sedation, defined as a MOAA/S ≤ 1 within 5 min of the first intravenous injection of fospropofol disodium (no use of propofol for rescue within 5 min).

Secondary outcomes: (1) time to onset of sedation; (2) Awakening time, defined as the time to open the eyes after the operation; (3) the number of additional times of fospropofol disodium during surgery; (4) Total dosage of fospropofol disodium;(5) the incidence of hypotension, which is defined as MAP <65 mmHg; (6) Vital signs (BP, HR, SpO_2_) at different times in routine clinical trials were monitored, and vital signs were recorded at time points after admission (T0), 1 min (T1), 3 min (T2), 5 min(T3) after anesthesia induction, and at the end of surgery (T4) to evaluate the impact and safety of drugs on respiration and circulation; (7) Occurrence of adverse reactions (injection pain, abnormal sensation/itching).

### 2.5 Statistical analysis

According to the results of the pre-test, the first sedation success rates of group P1, group P2 and group P3 were (6/10) 60%, (9/10) 90% and (10/10) 100%, respectively. Based on *α* = 0. 05, 1-*β* = 0.9, PASS 15 software was used to calculate the sample size, the total sample size was 61 cases, the ratio of P1 group, P2 group and P3 group was 1: 1: 1, 21 cases in each group, considering the 15% shedding rate, a total of 74 patients were included.

Data were analyzed using SPSS26.0. The obtained data were tested for normal distribution and homogeneity of variance. The mean ± standard deviation was used for the normal distribution of continuous data, one-way ANOVA was used for comparison between groups, LSD test was used for post-hoc multiple comparison between groups, and paired T-test was used for intra-group comparison. Non-normally distributed continuous variables were expressed as median (interquartile range) [M(Q1 ~ Q3)], and rank-sum test was used for comparison between groups. Count data were expressed as percentages, chi-square test was used for comparison between groups, and *p* values were adjusted using the Bonferroni method for post-hoc multiple comparisons between groups. The level of statistical significance for all of the above tests is defined as a probability value of less than 0.05.

## 3 Results

### 3.1 Patient condition

**Figure 1:**
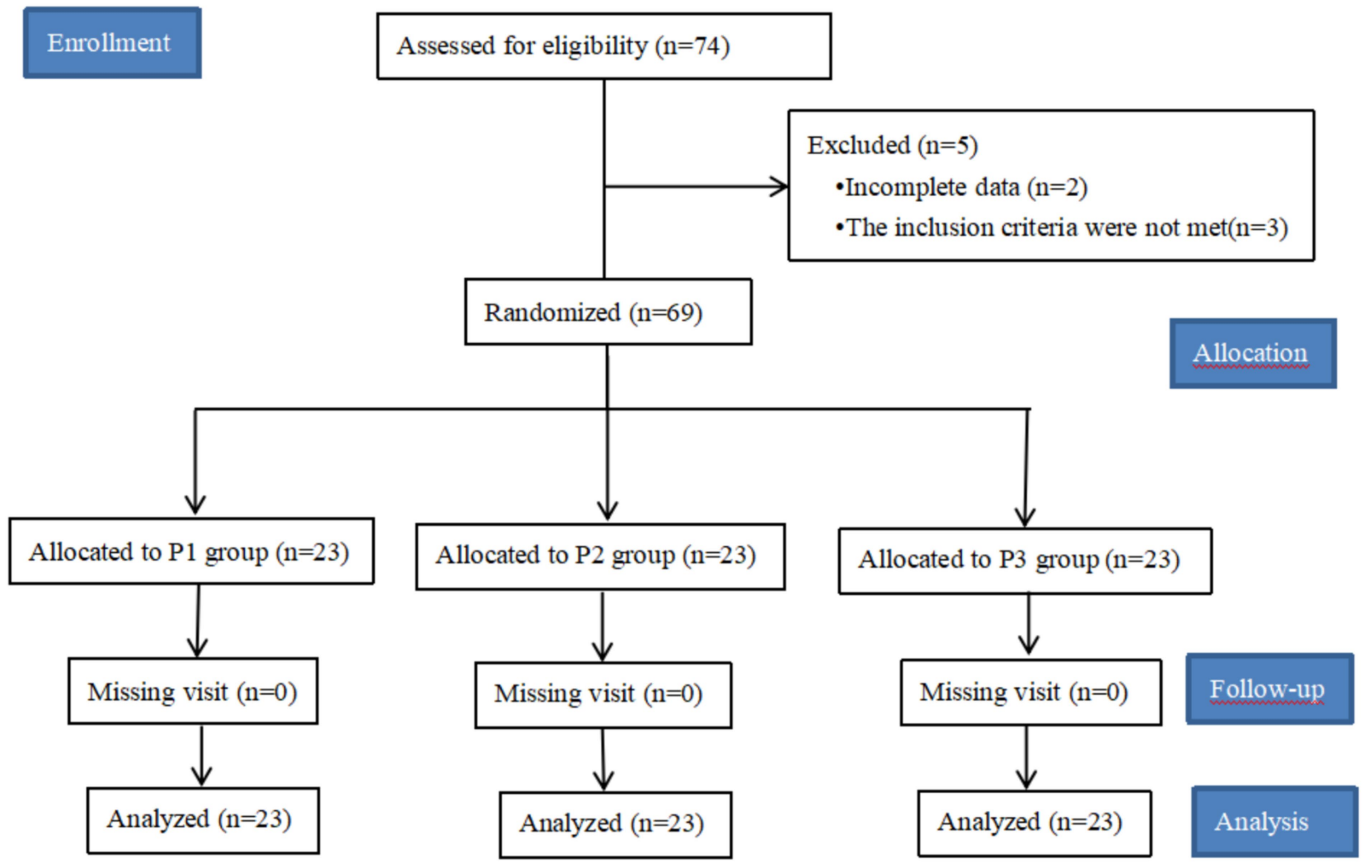
Research flowcharts. A total of 74 patients were included in this study, but the data of 2 patients were missing, and the remaining 3 patients did not meet the inclusion criteria and were excluded. Of the 69 patients collected, no patients were lost to follow-up, a total of 69 patients were included in the data analysis.

A total of 74 patients were included in this study, 5 cases were excluded, of which 2 patients were excluded due to incomplete key data records, 2 patients had more than 30 min of operation time, 1 patient had a BMI of >30 kg/m^2^, and 69 patients were finally included in this study. See Figure 1.

Comparison of general condition and operation time: There was no statistical difference in general information and operation time among the three groups (*p* > 0.05), see Table 1.

**Table 1:** The general condition and operation time of the three groups were compared.

Variables	P1 Group (*n* = 23)	P2 Group (*n* = 23)	P3 Group (*n* = 23)	*p* value
Age (years)	47.00 ± 10.98	49.30 ± 8.79	47.34 ± 11.59	0.191
BMI(kg/m^2^)	24.77 ± 2.34	24.38 ± 3.97	23.58 ± 4.86	0.466
ASA				0.538
I	4 (17.4)	3 (13.0)	5 (21.7)	
II	19 (82.6)	20 (87.0)	18 (78.3)	
Duration of surgery (min)	18.00 (12.00, 25.00)	17.00 (13.00, 23.00)	19.00 (15.00, 27.00)	0.088

Data are expressed as Means ± standard deviations, absolute rate, and percentage. Statistical significance was defined as *p* < 0.05. BMI, body mass index; ASA, American Society of Anesthesiologists.

### 3.2 Primary and secondary outcomes

The success rate of first sedation in the P2 and P3 groups was significantly higher than that in the P1 group (P1 vs. P2 vs. P3 = 52.2% vs. 91.3% vs. 100%; *p* < 0.05), and there was no difference between P2 group and P3 group (*p* > 0.05). The onset time of sedation was significantly shorter in the P2 and P3 groups than in the P1 group (*p* < 0.05), and there was no difference between the P2 group and the P3 group (*p* > 0.05). The awakening time of the P2 and P3 groups was significantly longer than that of the P1 group (*p* < 0.05), and there was no difference between the P2 group and the P3 group (*p* > 0.05), see Table 2.

**Table 2:** Primary and secondary observational indicators.

Variables	P1 Group (*n* = 23)	P2 Group (*n* = 23)	P3 Group (*n* = 23)	*p* value
First sedation success rate	12 (52.2)	21 (91.3)^a^	23 (100)^a^	0.001
The time to onset of sedation (min)	4.60 (4.10, 5.00)	2.00 (2.00, 4.00)^a^	2.00 (2.00, 3.00)^a^	0.001
The time of awakening (min)	5.00 (2.00, 6.00)	9.00 (6.00, 12.00)^a^	7.00 (4.00, 15.00)^a^	0.001
Intraoperative additions of fospropofol disodium	1 (0, 1)	0 (0, 1)	0 (0, 1)	0.221
Total dosage of fospropofol disodium (mg)	771.30 ± 109.6	944.39 ± 162.55	1093.69 ± 188.60	0.001
Hypotension	1 (4.3)	2 (8.7)	9 (39.1) ^ab^	0.003

Data are expressed as M ± SD, median (interquartile range), or number (%). Statistical significance was defined as *p* < 0.05. Compared with the P1 group, the ^a^*p*<0.05; Compared with the P2 group, the ^b^*p*<0.05.

There was no difference in the number of intraoperative additions of fospropofol disodium among the three groups (*p* > 0.05), There were differences in the total dosage of fospropofol disodium among the three groups.(P1 vs. P2 vs. P3 = 771.30 ± 109.6 mg vs. 944.39 ± 162.55 mg vs. 1093.69 ± 188.60 mg; *p* < 0.05), see Table 2.

Compared with the P1 and P2 groups, the incidence of hypotension in the P3 group was significantly higher (P1 vs. P2 vs. P3 = 4.3% vs. 8.7% vs. 39.1%; *p* < 0.05), and there was no difference in the incidence of hypotension between the P1 and P2 groups (*p* > 0.05), see Table 2.

### 3.3 Comparison of patient monitoring indicators

In the P1 group, compared with T0, there was no difference in MAP and HR at T1 and T2 (*p* > 0.05). MAP and HR were significantly decreased at T3 (*p* < 0.05). In the P2 group, the MAP at T1, T2 and T3 was significantly decreased (*p* < 0.05), the HR at T1 and T2 was not different (*p* > 0.05), and the HR at T3 was significantly decreased. In the P3 group, MAP and HR at T1, T2 and T3 were significantly decreased (*p* < 0.05). At T2, the MAP of the P3 group decreased significantly compared with the P1 and P2 groups (*p* < 0.05), and there was no significant MAP between the P1 and P2 groups (*p* > 0.05). There were no differences in MAP, HR and SpO_2_ at the remaining time points among the three groups (*p* > 0.05), see Table 3.

**Table 3:** Comparison of patient monitoring indicators.

Variables	Group	T0	T1	T2	T3
MAP	P1	100.39 ± 17.58	95.52 ± 12.80	92.61 ± 13.38	83.65 ± 13.55^a^
	P2	102.04 ± 13.67	91.17 ± 12.32^a^	85.56 ± 15.83^a^	81.22 ± 16.51^a^
	P3	95.17 ± 11.22	87.17 ± 11.73^a^	76.17 ± 14.79^abc^	75.22 ± 13.36^a^
HR	P1	72.75 ± 21.52	69.75 ± 20.37	66.75 ± 16.36	64.50 ± 18.52^a^
	P2	69.55 ± 10.79	66.75 ± 9.40	68.20 ± 9.85	64.65 ± 9.50^a^
	P3	72.22 ± 11.18	67.22 ± 8.08^a^	67.22 ± 8.08^a^	67.22 ± 9.12^a^
SpO_2_	P1	100.00 (99.00, 100.00)	98.00 (96.00, 100.00)	97.00 (95.00, 100.00)	97.00 (95.00, 100.00)
	P2	98.00 (96.00, 100.00)	97.00 (95.00, 100.00)	97.00 (94.00, 100.00)	98.00 (96.00, 100.00)
	P3	99.00 (97.00, 100.00)	98.00 (97.00, 100.00)	99.00 (98.00, 100.00)	97.00 (95.00, 100.00)

Data are expressed as M ± SD, median (interquartile range). Compared with T0, the ^a^*p* < 0.05.T2 time point: Compared with the P1 group, the ^b^*p* < 0.05; Compared with the P2 group, the ^c^*p* < 0.05.

### 3.4 Occurrence of adverse reactions

There was no difference in the occurrence of injection pain among the three groups (*p* > 0.05). There was no difference in the incidence of abnormal sensation/itching among the three groups (*p* > 0.05), see Table 4.

**Table 4:** Occurrence of adverse reactions between groups.

Variables	P1 (*n* = 23)	P2 (*n* = 23)	P3 (*n* = 23)	Total	*p* value
Injection pain	1 (4.3)	2 (8.7)	1(4.3)	4 (5.8)	0.767
Abnormal sensation/itching	14 (60.9)	10 (43.5)	13 (56.5)	37 (53.6)	0.469

Data are expressed as number (%). Statistical significance was defined as *p* < 0.05.

## 4 Discussion

Cohen (8) evaluated the sedative effect of fospropofol disodium for colonoscopy in a phase III clinical trial. The results showed that the success rate of sedation with 8 mg/kg fospropofol disodium (MOAA/S score < 4) was 96%. Li Jinhui’s (9) found that used fospropofol disodium 10 ~ 12.5 mg/kg combined with other drugs for general anesthesia induction of adult tracheal intubation, and the success rate of sedation (MOAA/S score ≤ 1) was 100%. In the phase III clinical trial, only 20 mg/kg of fospropofol disodium was used to evaluate the sedative effect. The success rate of sedation (no additional drugs within 5 min, MOAA/S score ≤ 1) was 97.7% (10). Considering that our painless colonoscopy diagnosis and treatment combined with low-dose alfentanil 5 μg/kg, this study designed three different doses of 10 mg/kg, 12.5 mg/kg, and 15 mg/kg of fospropofol disodium for painless colonoscopy diagnosis and treatment of deep sedation.

This study confirmed that the success rate of the first sedation of 12.5 mg/kg and 15 mg/kg of fospropofol disodium in painless colonoscopy was relatively high, which was similar to the success rate of fospropofol disodium 20 mg/kg for general anesthesia induction in endotracheal intubation in phase III clinical trial (10). Another study by Cohen et al. (11) found that the success rate of MAC sedation in patients with fospropofol disodium 6.5 mg/kg was 87%. Silvestri et al. (12) found that 6.5 mg/kg fospropofol disodium for bronchoscopy provided satisfactory sedation, safety and efficacy. The results of this study showed that the success rate of sedation was higher than that of the two studies, which may be related to the combination of 5 μg/kg of alfentanil and the different definition of the success rate of sedation. This study showed that the onset time of sedation in painless colonoscopy with 12.5 mg/kg and 15 mg/kg of fospropofol disodium was faster than that of 10 mg/kg, and the recovery time was prolonged. This may be that with the increase of dosage, the onset time of sedation or anesthesia is faster, the duration is gradually prolonged, and the recovery time is also increased in a dose-dependent manner (6).

The results of this study showed that there was no significant difference in the number of additional doses of propofol disodium between the three groups, but the total amount of fospropofol disodium was different, which may be related to the different doses of the first injection. For colonoscopy diagnosis and treatment with a long expected time, the traditional anesthetic sedative propofol single dose cannot meet the needs of surgery, and continuous pumping is often required during the operation. However, the half-life of fospropofol disodium is long, and it can be administered in a single dose, which is more convenient to use and can reduce the risk of propofol fat emulsion infusion-related infection (13).

The results of this study showed that different doses of fospropofol disodium injection, will produce different degrees of hemodynamic changes, with the increase of the dose, the greater the hemodynamic changes, which may be related to the increase of the depth of sedation after the increase of the dose. However, the decrease of MAP and HR in most patients is within 30% of the basic value, and 200–300 mL of fluid can be rapidly replenished before administration to reduce its effect on hemodynamics (6). Compared with the three groups at each time point, the P3 group had a greater effect on hemodynamics at T2 time point than the other two groups, indicating that the combination of 5 μg/kg of alfentanil and 15 mg/kg of fospropofol disodium had a greater impact on hemodynamics during painless colonoscopy. It is recommended to strengthen monitoring in clinical practice. Secondly, there was no significant change in SpO_2_ in the three groups during the whole anesthesia process, indicating that fospropofol disodium had a slight effect on respiration during sedation and had good safety, which was consistent with the results of Atlas et al. (14, 15).

Euasobhon et al. (16) showed that the incidence of high-intensity pain in intravenous propofol was 38.1%, which significantly reduced patient comfort and satisfaction. In this study, we found that the incidence of injection pain of fospropofol disodium was lower than that of propofol, which may be related to its water-soluble and fat-free emulsion (17). This study found that the main adverse reaction of fospropofol disodium is abnormal sensation / itching, the incidence rate is 53.6%, but the clinical observation is mild and self-limited, generally lasting 1 ~ 2 min. It has been reported that the incidence of abnormal sensation / pruritus after a single injection of fospropofol disodium can reach 62% ~ 95% (10, 18), which may be related to the increase of phosphate level (19). Previous studies have shown that opioid receptors may be involved in the occurrence of itching, and interventions targeting opioid receptors can reduce itching symptoms (20). In this study, a small dose of alfentanil was used before the injection of propofol disodium, which could reduce the incidence of abnormal sensation /itching to a certain extent. Zhang et al. (21) found that nalbuphine can significantly reduce the incidence of abnormal sensation/pruritus after extubation of general anesthesia with fospropofol sodium. The incidence of abnormal sensation / pruritus was 24.5%, which was much lower than that in this study. The incidence of abnormal sensation/pruritus may be related to different observation time points.

There are also shortcomings in this study: this study is a dose exploratory study and is not compared with the classical sedative propofol. The sample size is small, and further large-sample, multi-center clinical studies are needed. The appropriate dose of alfentanil to prevent abnormal sensation/itching caused by propofol disodium needs to be further explored.

## 5 Conclusion

In conclusion, the combination of alfentanl 5 μg/kg and fospropofol disodium 12.5 mg/kg has a high success rate of the first sedation and a small impact on hemodynamics, which has certain clinical advantages.

## Data Availability

The raw data supporting the conclusions of this article will be made available by the authors, without undue reservation.
